# Radiomics analysis of lesion-specific pericoronary adipose tissue to predict major adverse cardiovascular events in coronary artery disease

**DOI:** 10.1186/s12880-024-01325-1

**Published:** 2024-06-17

**Authors:** Meng Chen, Guangyu Hao, Jialiang Xu, Yuanqing Liu, Yixing Yu, Su Hu, Chunhong Hu

**Affiliations:** 1 Department of Radiology, The First Affiliated Hospital of Soochow University, NO.899 Pinghai Road, Gusu District, Suzhou, Jiangsu 215006 China; 2 Department of Cardiology, The First Affiliated Hospital of Soochow University, NO.899 Pinghai Road, Gusu District, Suzhou, Jiangsu 215006 China

**Keywords:** Radiomics, Lesion-specific, Pericoronary adipose tissue, Major adverse cardiovascular events

## Abstract

**Objective:**

To investigate the prognostic performance of radiomics analysis of lesion-specific pericoronary adipose tissue (PCAT) for major adverse cardiovascular events (MACE) with the guidance of CT derived fractional flow reserve (CT-FFR) in coronary artery disease (CAD).

**Materials and methods:**

The study retrospectively analyzed 608 CAD patients who underwent coronary CT angiography. Lesion-specific PCAT was determined by the lowest CT-FFR value and 1691 radiomic features were extracted. MACE included cardiovascular death, nonfatal myocardial infarction, unplanned revascularization and hospitalization for unstable angina. Four models were generated, incorporating traditional risk factors (clinical model), radiomics score (Rad-score, radiomics model), traditional risk factors and Rad-score (clinical radiomics model) and all together (combined model). The model performances were evaluated and compared with Harrell concordance index (C-index), area under curve (AUC) of the receiver operator characteristic.

**Results:**

Lesion-specific Rad-score was associated with MACE (adjusted HR = 1.330, *p =* 0.009). The combined model yielded the highest C-index of 0.718, which was higher than clinical model (C-index = 0.639), radiomics model (C-index = 0.653) and clinical radiomics model (C-index = 0.698) (all *p* < 0.05). The clinical radiomics model had significant higher C-index than clinical model (*p* = 0.030). There were no significant differences in C-index between clinical or clinical radiomics model and radiomics model (*p* values were 0.796 and 0.147 respectively). The AUC increased from 0.674 for clinical model to 0.721 for radiomics model, 0.759 for clinical radiomics model and 0.773 for combined model.

**Conclusion:**

Radiomics analysis of lesion-specific PCAT is useful in predicting MACE. Combination of lesion-specific Rad-score and CT-FFR shows incremental value over traditional risk factors.

**Supplementary Information:**

The online version contains supplementary material available at 10.1186/s12880-024-01325-1.

## Introduction

Coronary artery disease (CAD) is the leading cause of death threatening human health and presents a significant economic burden [1], and the progression of CAD is asymptomatic, following a severe clinical course occasionally [2]. To date, coronary computed tomography angiography (CCTA) is a primary-line noninvasive modality for the diagnosis and assessment of CAD [3], which mainly detects coronary artery calcium, stenosis rate and plaque characteristics. Vascular inflammation has long been demonstrated a central driver of coronary atherosclerosis development and plaque rupture [4], resulting in acute coronary syndrome (ACS), which is the leading cause of CAD in the world [5]. Circulating inflammatory biomarkers, such as C-reactive protein, are inadequately specific. Positron emission tomography-CT is expensive and not commonly accessible, though it can detect coronary inflammation [6].

Inflamed coronaries release mediators which can contribute to histopathological changes of pericoronary adipose tissue (PCAT), leading to increased edema and decreased adipocyte size, because there exists bidirectional interplay between them [7]. CRISP-CT study [8] and other research [9] pointed out PCAT CT attenuation (PCATa) around right coronary artery (RCA) had incremental prognostic value beyond clinical characteristics, qualitative plaque features and quantitative plaque parameters. Furthermore, structural changes in PCAT, including fibrosis and microvascular remodeling, are caused by chronic atherosclerosis and inflammation [10] and can be captured by radiomics analysis [11]. CCTA-based radiomics analysis of PCAT showed advantages in discriminating acute myocardial infarction (MI) from unstable angina [4] and predicting cardiac risk [11].

Previous researches have demonstrated PCAT analysis surrounding coronary plaques to be a potential sensor of plaque vulnerability [12]. Our preliminary study found lesion-specific inflammation preceded global inflammation in prognostic evaluation [13]. Furthermore, CT-derived fractional flow reserve (CT-FFR) has shown high accuracy to detect functional myocardial ischemia with invasive FFR as the gold standard [14, 15]. According to the criteria of Society of Cardiovascular Computed Tomography (SCCT) [16], CT-FFR was recommended to assess lesion-specific ischemia. Therefore, this study aims to explore the prognostic performance of radiomics analysis of lesion-specific PCAT for major adverse cardiovascular events (MACE) with the guidance of CT-FFR.

## Materials and methods

### Study population

Local institutional review board and the ethics committee approved this retrospective study and waived the written informed consent. 608 CAD patients were included in this study and the exclusion criteria were shown in the Supplementary Material and Fig S1. Patients were divided with a ratio of 7:3 using a random number table (Supplementary Material).

Clinical risk factors were collected, including age, sex, hypertension (systolic blood pressure ≥ 140 mmHg or diastolic blood pressure ≥ 90 mmHg or taking antihypertensive drugs), hyperlipidemia (serum total cholesterol ≥ 230 mg/dl or triglyceride ≥ 200 mg/dl or taking lipid-lowering drugs), diabetes [(1) typical diabetes symptoms + (2) fasting blood glucose level ≥ 7mmol or random blood glucose level ≥ 11.1 mmol or oral glucose tolerance test ≥ 11.1 mmol or taking hypoglycemic drugs), history of smoking or drinking (having a history of smoking/drinking at present or in the past one year). In addition, medication and planned revascularization therapy within 60 days were also acquired.

### CCTA protocols

Image acquisitions and reconstruction were presented in our previous study [13] and provided in the Supplementary Material and Table S1.

### Coronary plaque analysis

Coronary artery calcium score (CACS) was measured on the basis of the Agatston score [17]. Qualitative plaque analyses and quantitative plaque parameters were processed by an onsite software (CoronaryDoc®, ShuKun Network Technology). Diameter stenosis (DS) was divided into: minimal stenosis, mild stenosis, moderate stenosis, severe stenosis and occlusion [16]. Coronary plaque was classified as calcified, noncalcified or mixed plaque on per-plaque basis [18]. High-risk plaque (HRP) was regarded as plaque with two or more high-risk features (low attenuation, spotty calcification, napkin ring sign and positive remodeling) [16, 19]. Segment involvement score (SIS) was defined as involved segments according to the 18 segments criteria of SCCT [20]. Plaque volumes were the total volume of calcified (>350 HU), fibrous (30–350 HU) and lipid (<30 HU) plaque.

### Quantification measurement of CT-FFR and identification of target plaque

CT-FFR was calculated by an automated software (CoronaryDoc®-FFR, Shukun Technology, Beijing, China). The 3D geometrical morphology of the coronary artery was constructed with the segmented arteries and localized arteries center lines from the conventional standardized CCTA image data. The entrance, exit and boundary conditions of the hemodynamic model were then determined from the geometrical morphology of the artery. CT-FFR value at any position of the coronary artery was obtained from the reduced-order computational fluid dynamics model [2]. Lesion-specific CT-FFR was measured at 2 cm away from the plaque. CT-FFR ≤ 0.8 was defined as hemodynamically significant stenosis.

**Fig. 1:**
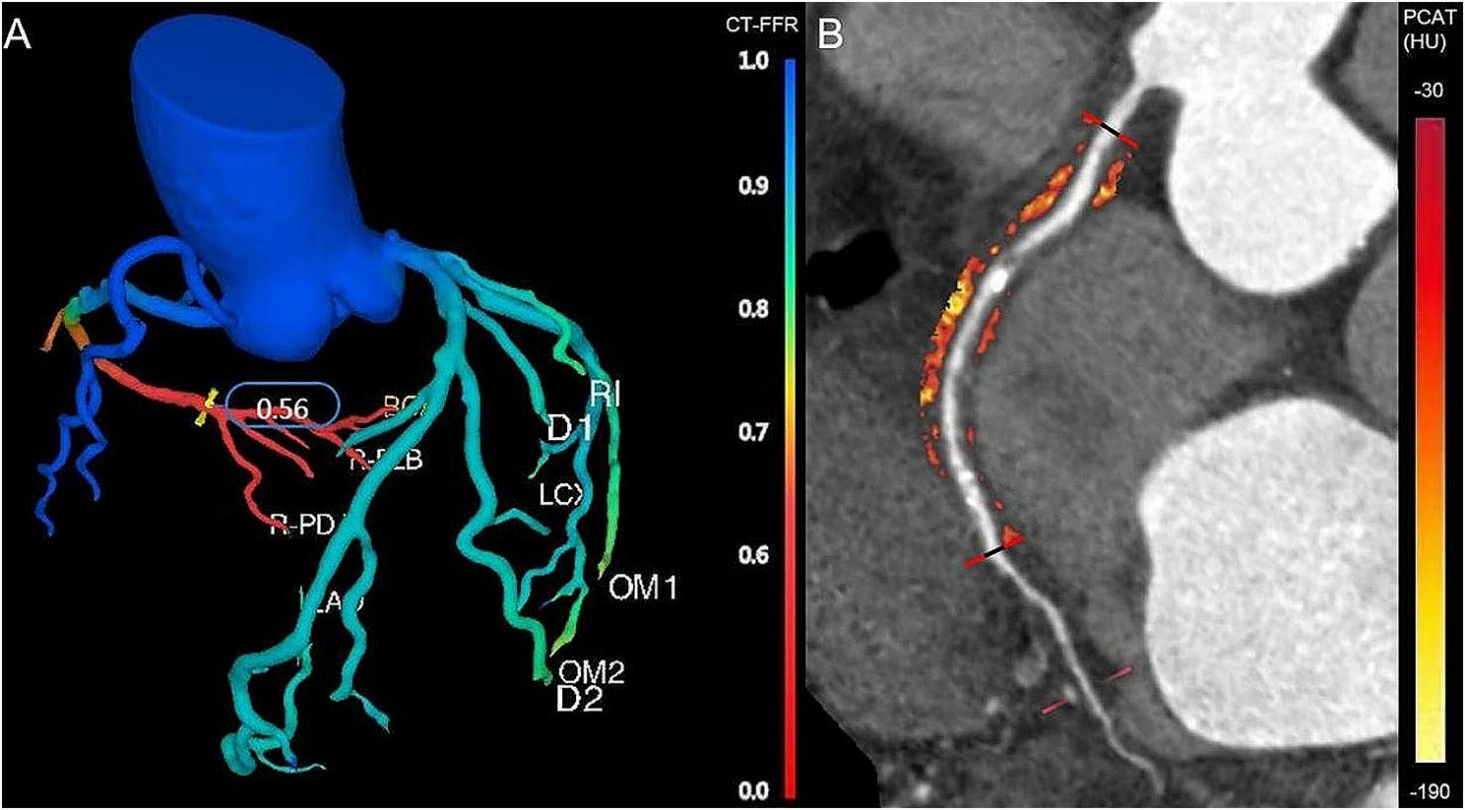
An example of target plaque (RCA, 1 A and 1B) and corresponding lesion-specific PCAT (1B). The black and red line (1B) represented the range of target plaque and PCAT within a radial distance equal to the diameter of the vessel. RCA, right coronary artery; PCAT, pericoronary adipose tissue

Definite plaque was firstly detected in left main coronary artery (LM), left anterior descending artery (LAD), left circumflex artery (LCX) and RCA. Target plaque was determined based on the most significant hemodynamic lesion (the lowest CT-FFR value) in the patient-based analysis. Figure 1 showed an example of target plaque and corresponding lesion-specific PCAT.

### Segmentation and radiomics extraction of lesion-specific PCAT

A dedicated PCAT module in CoronaryDoc® was used for semi-automated quantification of lesion-specific PCAT. PCAT was regarded as the adipose tissue located within a radial distance from the outer vessel wall equal to the diameter of the vessel ranging from − 190 to -30 HU. The proximal and distal positions of target plaque were delineated manually and lesion-specific PCAT was determined in a semi-automated manner by tracking the contour of coronary artery. Moreover, 1691 radiomic features were extracted automatically from lesion-specific PCAT, including first-order, shape and texture features on the original images, and high-order features describing filter images features, which were as follow: (1) first-order statistics: the intensity features containing gray histogram information; (2) shape features: the size and shape information of PCAT; (3) texture features: these features measured the relationship between each PCAT voxel and its surrounding environments, including Gray Level Dependence Matrix (GLDM) features, Gray Level Co-occurrence Matrix (GLCM) features, Gray Level Run Length Matrix (GLRLM) features, Gray Level Size Zone Matrix (GLSZM) features and Neighbouring Gray Tone Difference Matrix (NGTDM) features; (4) higher-order features: wavelet transform images, nonlinear strength transformation of image voxel and local binary patterns (LBP) [21]. Wavelet transform images were generated by 8 different combinations of high and low frequency bands in 3 directions (x, y, z), providing high-dimensional multi-frequency information which were difficult to be visually interpreted. Nonlinear strength transformation of image voxel included square, square root, logarithm, exponential and gradient operations. LBP were computed two- and three-dimensionally and extracted with four variants, in which three-dimensional method used a level of one and two, as well as the kurtosis image. A radiologist (reader 1) with 10 years of experience in cardiac imaging delineated manually the range of target plaque and extracted the radiomics features of lesion-specific PCAT.

### Intra- and inter-observer consistency

CCTA plaque parameters, lesion-specific CT-FFR and radiomics features of lesion-specific PCAT were assessed by the radiologist (reader 1). 50 random patients were selected to evaluate the variabilities of all imaging parameters by two radiologists (reader 1; reader 2, with 11 years of experience in cardiac imaging). The same observer evaluated the imaging parameters after 1 month interval. The intra- and inter-class correlation coefficients (ICC) were calculated. For radiomics features, only features with ICC both intra- and inter-observer ICCs >0.8 were selected for further analysis.

### Radiomics features selection and model construction

**Fig. 2:**
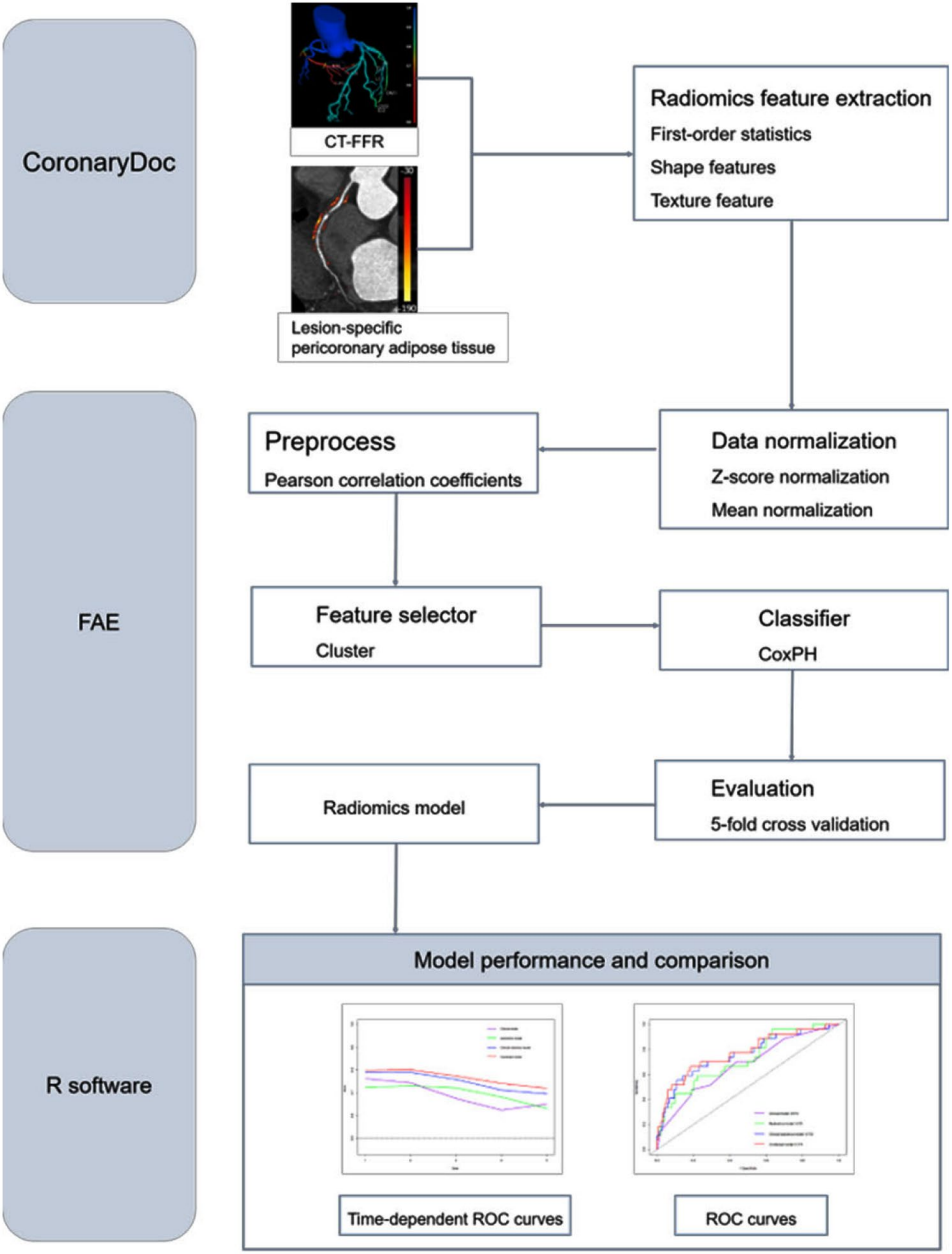
The procedure of radiomics features selection and model establishment

An open-source free application, FeAture Explorer Pro (FAE, version 0.5.5; http://github.com/salan668/FAE) [22], was used to analyze all the radiomics features and construct radiomics model. Data normalization (two methods), dimension reduction [Pearson correlation coefficients (PCC)], feature selection algorithms (cluster analysis) and classifier [Cox proportional hazards (CoxPH) regression] were used to select the useful radiomics features. The radiomics model was built based on the radiomics score (Rad-score) through a linear combination of selected features weighted by their regression coefficients derived from the CoxPH regression. Rad-score = $\sum _{i=1}^{n}\beta i Xi$, *Xi* was the feature selected by CoxPH regression and *βi* was the corresponding regression coefficient. A 5-fold cross-validation in the training cohort was used to determine the candidate combinations of the selected features. The radiomics model with greatest performance in the validation cohort would be selected as the final model. The procedure of features selection and radiomics model establishment was shown in Fig. 2.

Based on the training cohort, traditional risk factors including clinical risk factors and plaque parameters, Rad-score and CT-FFR were filtered first by univariate Cox proportional hazards regression and variables with *P <* 0.05 were incorporated into multivariate Cox regression. Independent predictors with *P <* 0.05 in multivariate Cox regression were used to establish clinical (traditional risk factors), radiomics (Rad-score), clinical radiomics (traditional risk factors and Rad-score) and combined (all together) models.

### Follow-up and MACE

Patients were followed up until December 2022. MACE included cardiovascular death, nonfatal MI, unplanned revascularization and hospitalization because of unstable angina (Supplementary Material) [23, 24]. An experienced cardiologist evaluated MACE independently.

### Statistical analysis

There existed missing data among 2.3% of patients, and the involved categorical variables were imputed with the mode value. SPSS Statistics (v26.0) and R software (v4.05) were used for the statistical analysis. A two-sided *p <* 0.05 was considered statistically significant.

One-sample Kolmogorov-Smirnov test was used to check if the quantitative variable was normally distributed. Quantitative variables with normal distribution were recorded as means ± standard deviations and compared with Independent-Sample *T* test, while median (quartiles) and Mann-Whitney *U* test were used otherwise. Categorical parameters were expressed with count (%) and analyzed with chi-square test. The intra- and inter-observer reliability for imaging parameters was assessed by ICC or kappa statistic.

Patients were separated into high-risk and low-risk subgroups according to the optimal cut-point of Rad-score in the training cohort, which was determined by “Survminer” package. “Survival” and “Survminer” packages were used to draw the Kaplan-Meier curves, which were compared by using the log-rank test. Harrell concordance index (C-index) and time-dependent area under receiver operating characteristic (ROC) curves (AUC) were used to evaluate model performance. “CsChange” package was used to calculate Δ C-index and statistical significance [25] and the bootstrap method of 200 replications was used to compute 95% CIs. “TimeROC” package was used to analyze the time-dependent ROC curves.

## Results

### Clinical characteristics

Table 1 showed the clinical characteristics of 608 patients. Median follow-up period was 58 (48, 75) months and146 patients were verified MACE. 3.1% of patients (*n* = 19) were judged as censored because they were lost, in which 13 and 6 patients were in the training and validation cohort respectively. Clinical characteristics and plaque features between patients with and without MACE were compared in the training and validation cohorts (Table 1). MACE occurrence rates between the two cohorts had not significant differences (*p* = 0.991). Patients with MACE had higher percentages of male, moderate and above stenosis, mixed plaque, HRP, β-blocker use and had higher CACS, SIS and plaque volumes than patients without MACE in the training and validation cohorts (all *p* < 0.05). Other variables showed discrepancies or no significant association with MACE in both cohorts.

**Table 1:** Clinical characteristics, plaque parameters and therapy between patients with and without MACE in the training and validation cohorts

Characteristics [median (quartiles) or *n* (%]	Training cohort (*n* = 425)			Validation cohort (*n* = 183)		
	MACE (*n* = 102)	No MACE (*n* = 323)	*P* value	MACE (*n* = 44)	No MACE (*n* = 139)	*P* value
Age	65 (57, 72)	62 (56, 70)	0.140	68 (59, 75)	63 (56,70)	0.026*
Male	80 (78.4)	193 (59.8)	0.001*	34 (77.3)	84 (60.4)	0.042*
Risk factors						
Hypertension	80 (78.4)	221 (68.4)	0.053	35 (79.5)	91 (65.5)	0.079
Hyperlipidemia	59 (57.8)	184 (57.0)	0.876	24 (54.5)	77 (55.4)	0.921
Diabetes mellitus	41 (40.2)	84 (26.0)	0.006*	11 (25.0)	34 (24.5)	0.942
Smoking	45 (44.1)	89 (27.6)	0.002*	20 (45.5)	45 (32.4)	0.114
Drinking	27 (26.5)	53 (16.4)	0.023*	9 (20.5)	37 (26.6)	0.411
Diameter stenosis			<0.001*			<0.001*
1–49%	11 (10.8)	141 (43.7)		5 (11.4)	58 (41.7)	
50–69%	30 (29.4)	86 (26.6)		13 (29.5)	35 (25.2)	
70–99%	46 (45.1)	89 (27.6)		20 (45.5)	43 (30.9)	
100%	15 (14.7)	7 (2.2)		6 (13.6)	3 (2.2)	
Plaque location			0.007*			0.435
LM	33 (11.1)	59 (8.4)		18 (14.4)	36 (10.8)	
LAD	101 (33.9)	321 (45.6)		43 (34.4)	135 (40.7)	
LCX	64 (21.5)	119 (16.9)		22 (17.6)	65 (19.6)	
RCA	100 (33.6)	205 (29.1)		42 (33.6)	96 (28.9)	
Plaque composition			<0.001*			0.010*
Non-calcified plaque	50 (16.8)	206 (29.3)		24 (19.2)	80 (24.1)	
Calcified plaque	70 (23.5)	202 (28.7)		21 (16.8)	91 (27.4)	
Mixed plaque	178 (59.7)	296 (42.0)		80 (64.0)	161 (48.5)	
High-risk plaque	68 (66.7)	103 (31.9)	<0.001*	21 (47.7)	52 (37.4)	0.223
CACS	92.30 (14.05, 371.42)	40.81 (1.03, 177.71)	<0.001*	154.60 (8.26, 876.94)	55.69 (3.16, 207.40)	0.021*
SIS	6 (2, 8)	3 (2,5)	<0.001*	6 (3, 9)	3 (2, 6)	0.001*
Plaque volumes	161.77 (82.99, 284.99)	68.60 (30.07, 148.95)	<0.001*	156.33 (88.54, 342.99)	90.79 (41.83, 145.81)	<0.001*
Planned revascularization	36 (35.3)	75 (23.2)	0.016*	13 (29.5)	35 (25.2)	0.556
CABG	2 (2.0)	4 (1.2)		0 (0.0)	3 (2.2)	
PCI	33 (32.4)	71 (22.0)		11 (25.0)	32 (23.0)	
PTCA	1 (1.0)	0 (0.0)		2 (4.5)	0 (0.0)	
Medication compliance						
Antiplatelet	86 (84.3)	248 (76.8)	0.106	35 (79.5)	109 (78.4)	0.873
Statin	89 (87.3)	245 (75.9)	0.014*	36 (81.8)	111 (79.9)	0.775
ACEI or ARB	78 (76.5)	212 (65.6)	0.040*	34 (77.3)	92 (66.2)	0.166
β-blocker	46 (45.1)	107 (33.1)	0.028*	21 (47.7)	43 (30.9)	0.042*
Antidiabetic	35 (34.3)	76 (23.5)	0.031*	10 (22.7)	29 (20.9)	0.792
Vasodilator	31 (30.4)	44 (13.6)	<0.001*	13 (29.5)	24 (17.3)	0.077

* represents *P* < 0.05

LM, left main coronary artery; LAD, left anterior descending artery; LCX, left circumflex artery; RCA, right coronary artery; CACS, coronary artery calcium score; SIS, segment involvement score; CABG, coronary artery bypass grafting; PCI, percutaneous coronary intervention; PTCA, percutaneous transluminal coronary angioplasty; ACEI, angiotensin converting enzyme inhibitor; ARB, angiotensin receptor blockers; MACE, major adverse cardiovascular events

Agreement for imaging parameters was excellent or good, which was observed in Supplementary Material Table S1.

### Construction and validation of the rad-score

**Fig. 3:**
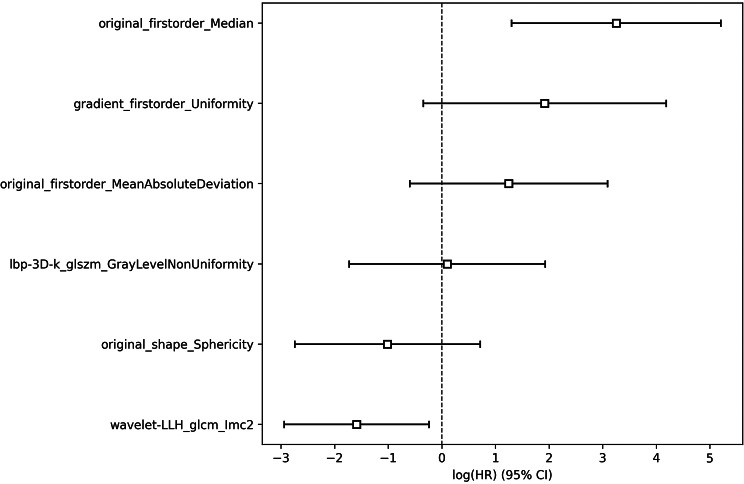
The regression coefficients of the selected features

After consistency, a total of 859 radiomics features were included in further analysis. The Mean data normalization, PCC dimension reduction, cluster feature selector and CoxPH classifier yielded the highest C-index with six features. The selected features were shown in Fig. 3 and Rad-score was calculated by using the following formula:

Rad-score = 3.242*original_firstorder_Median + 1.914*gradient_firstorder_Uniformity + 1.245*original_firstorder_MeanAbsoluteDeviation + 0.087*lbp-3D-k_glszm_GrayLevelNonUniformity-1.015*original_shape_Sphericity-1.585*wavelet-LLH_glcm_Imc2.

**Fig. 4:**
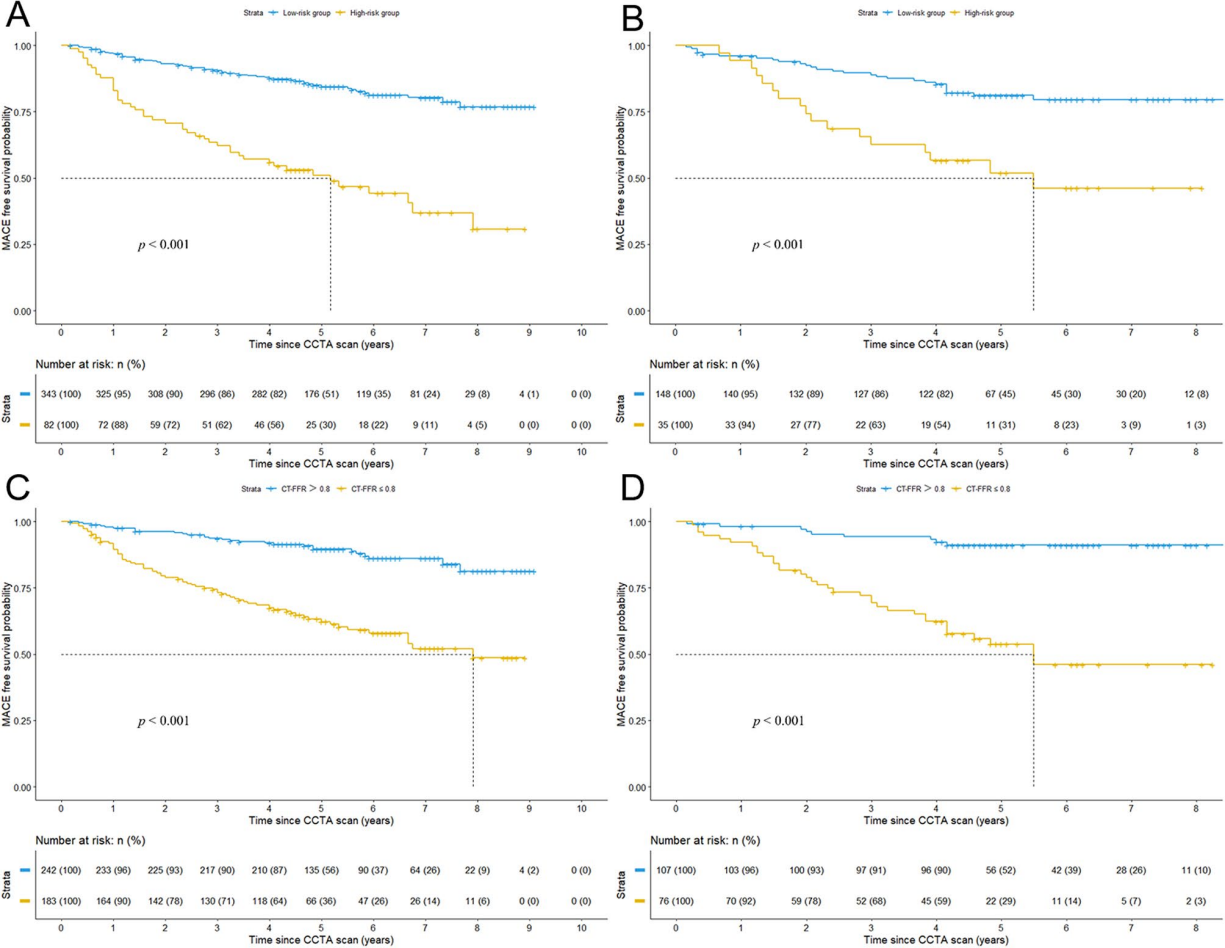
Kaplan-Meier curves between high-risk and low-risk groups, and CT-FFR ≤ 0.8 and CT-FFR > 0.8 groups in the training (**A**,**C**) and validation (**B**,**D**) cohorts. CT-FFR, CT derived fractional flow reserve

Rad-score was associated with MACE in the training cohort [adjusted HR = 2.064 (95% CI: 1.597–2.667), *p <* 0.001], which was confirmed in the validation cohort [adjusted HR = 1.330 (95% CI: 1.074–1.647), *p =* 0.009]. The optimum cutoff was 0.684 and patients were classified into high-risk group (Rad-score ≥ 0.684) and low-risk group (Rad-score < 0.684). Kaplan-Meier curve of high-risk group had significant decrease in MACE-free survival probability compared with low-risk group in the training and validation cohorts (Fig. 4a and b). Figure 4c and d depicted Kaplan-Meier curves between CT-FFR ≤ 0.8 group and CT-FFR > 0.8 group in the training and validation cohorts.

### Model establishment and performance

Univariate and multivariate Cox regression (Table 2) identified DS, HRP, Rad-score were independent predictors, which was used to construct clinical model and clinical radiomics model. Although CT-FFR was not independent predictor of MACE, lesion-specific PCAT was determined by CT-FFR. Thus, combined model was developed by combination of all independent predictors and CT-FFR. Multivariate Cox regression in the validation cohort (Table 3) demonstrated Rad-score and CT-FFR were associated with MACE.

**Table 2:** Univariate and multivariate Cox proportional hazard analysis of variables with MACE in the training cohort

Variable	Univariable analysis		Multivariable analysis	
	HR (95% CI)	*P* value	HR (95% CI)	*P* value
Male	2.144 (1.337–3.438)	0.002		0.414
Smoking	1.891 (1.279–2.796)	0.001		0.267
Drinking	1.656 (1.066–2.572)	0.025		0.470
Diabetes mellitus	1.739 (1.170–2.585)	0.006		0.300
CACS	1.000 (1.000-1.001)	0.027		0.832
Diameter stenosis (%)		<0.001		<0.001*
1–49	Reference		Reference	
50–69	4.174 (2.089–8.342)	<0.001	3.195 (1.588–6.428)	0.001
70–99	5.895 (3.050-11.392)	<0.001	3.341 (1.669–6.692)	0.001
100	15.072 (6.909–32.882)	<0.001	4.787 (2.038–11.245)	<0.001
High-risk plaque	3.452 (2.286–5.213)	<0.001	1.723 (1.087–2.733)	0.021*
SIS	1.238 (1.163–1.317)	<0.001		0.138
Total plaque volume	1.003 (1.002–1.004)	<0.001		0.829
Rad-score	2.708 (2.143–3.422)	<0.001	2.064 (1.597–2.667)	<0.001*
CT-FFR	0.004 (0.001–0.014)	<0.001		0.097

* represents *P* < 0.05

MACE, major adverse cardiovascular events; CACS, coronary artery calcium score; SIS, segment involvement score; Rad-score, radiomics score; CT-FFR, CT derived fractional flow reserve

**Table 3:** Multivariate Cox proportional hazard analysis of variables with MACE in the validation cohort

Variable	Multivariable analysis	
	HR (95% CI)	*P* value
Male		0.238
Smoking		0.154
Drinking		0.524
Diabetes mellitus		0.610
CACS		0.419
Diameter stenosis (%)		0.320
1–49	Reference	
50–69		0.464
70–99		0.916
100		0.326
High-risk plaque		0.508
SIS		0.553
Total plaque volume		0.689
Rad-score	1.330 (1.074–1.647)	0.009*
CT-FFR	0.010 (0.001–0.067)	<0.001*

* represents *P* < 0.05

MACE, major adverse cardiovascular events; CACS, coronary artery calcium score; SIS, segment involvement score; Rad-score, radiomics score; CT-FFR, CT derived fractional flow reserve

**Fig. 5:**
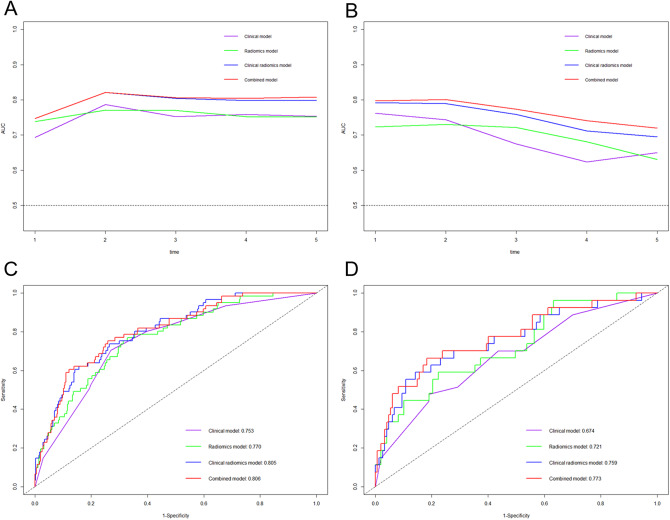
Plots of annual AUCs from 1–5 years and comparison of ROC curves (3 years) of the different models for discrimination of major adverse cardiovascular events in the training (**A**, **C**) and validation (**B**, **D**) cohorts. AUC, area under the receive operating characteristic curve (ROC)

Model performance and comparison were shown in Tables 4 and 5. In the validation cohort, the combined model yielded the highest C-index of 0.718, which was higher than clinical model (C-index = 0.639), radiomics model (C-index = 0.653) and clinical radiomics model (C-index = 0.698) (all *p* < 0.05). The clinical radiomics had significant higher C-index than clinical model (*p* = 0.030). There were no significant differences in C-index between clinical or clinical radiomics model and radiomics model (*p* values were 0.796 and 0.147 respectively). The time-dependent ROC revealed combined model had higher AUC than clinical radiomics, radiomics and clinical models in predicting MACE from 1 to 5 years (Fig. 5). In the validation cohort, the AUC increased from 0.674 for clinical model to 0.721 for radiomics model, 0.759 for clinical radiomics model and 0.773 for combined model at 3 years.

**Table 4:** Performance of clinical, radiomics, clinical radiomics and combined models for predicting MACE

Statistics indexes	Cohorts	Clinical model	Radiomics model	Clinical radiomics model	Combined model
C-index	Training	0.729 (0.683–0.776)	0.732 (0.684–0.780)	0.774 (0.731–0.817)	0.780 (0.736–0.823)
	Validation	0.639 (0.556–0.723)	0.653 (0.574–0.732)	0.698 (0.619–0.777)	0.718 (0.641–0.795)
Δ C-index (*P* value)	Training	1 (Reference)	0.003 (-0.051-0.054) (0.921)	0.045 (0.018–0.071) (<0.001)	0.050 (0.025–0.076) (<0.001)
	Validation		0.014 (-0.084-0.121) (0.796)	0.059 (0.008–0.113) (0.030)	0.078 (0.025–0.134) (0.004)
AUC	Training	0.753 (0.690–0.815)	0.770 (0.709–0.831)	0.805 (0.749–0.860)	0.806 (0.749–0.863)
	Validation	0.674 (0.562–0.786)	0.721 (0.616–0.827)	0.759 (0.652–0.866)	0.773 (0.669–0.878)
Δ AUC (*P* value)	Training	1 (Reference)	0.017 (0.972)	0.052 (0.016)	0.053 (0.018)
	Validation		0.047 (0.857)	0.085 (0.052)	0.099 (0.011)

MACE, major adverse cardiovascular events; C-index, Harrell concordance index; AUC, area under receiver operator characteristic curve

**Table 5:** Performance comparison among the radiomics, CT-FFR, clinical radiomics and combined models

Comparison among models	*P* value for C-index		*P* value for AUC	
	Training	Validation	Training	Validation
Clinical radiomics model vs. radiomics model	0.006	0.147	0.219	0.590
Combined model vs. radiomics model	0.005	0.024	0.237	0.340
Combined model vs. clinical radiomics model	0.192	0.008	0.999	0.085

C-index, Harrell concordance index; AUC, area under receiver operator characteristic curve

## Discussion

In this study, lesion-specific Rad-score was an independent predictor for MACE in CAD patients and had favorable predictive performance. Clinical radiomics model, which incorporate Rad-score and traditional risk factors, was superior to clinical model in MACE evaluation. Combined model, encapsulating traditional risk factors, Rad-score and CT-FFR, outperformed other models, which indicated lesion-specific Rad-score and CT-FFR showed incremental value over traditional risk factors.

Previous studies demonstrated lesion-specific radiomics had the potential to identify vulnerable plaques [26] and MI [27], detect functional myocardial ischemia [28] and predict the occurrence of subsequent ACS [29]. Different form above researches, the priority of the present study lies on the combination of radiomics analysis of target plaque and CT-FFR to predict MACE in CAD patients. Relevant study indicated radiomics features of PCAT improved CT-FFR performance in predicting hemodynamically significant CAD [30], which showed the CAD patients would benefit from the combination of PCAT radiomics and CT-FFR.

Current risk management depends on plaque parameters assessment, such as CACS, anatomical severity and HRP. The CACS can accurately estimate the severity of CAD [31] and predict cardiovascular events [32], however CACS is recommended for low-middle risk patients [33]. Our study demonstrated DS and HRP were independent predictors for MACE, which was in accord with previous researches indicating adverse coronary plaque characteristics and obstructive disease were associated with CAD death or nonfatal MI [34]. Furthermore, researches proved inflammation was an important driver of coronary plaque development and rupture [35], and PCATa was significantly associated with perivascular inflammation with 18 F-FDG PET as the reference standard. Oikonomous et al. indicated PCATa enhanced cardiac risk prediction [8], and further used radiomic profiles of PCAT to describe fibrosis and vascularity and improve risk prediction for adverse clinical events [11].

Majority of previous studies investigated radiomics analysis of PCAT in the proximal 40-mm segment of three major epicardial coronary vessels (LAD, LCX and RCA) [2, 4, 36] or one of them (RCA or LAD) [11, 27]. Different from previous researches, this research focused on radiomics analysis of lesion-specific PCAT, which was influenced directly by coronary plaque, highlighting the direct “cross-talk” between the coronary arterial wall and adjacent pericoronary adipocytes [37]. Some studies have demonstrated lesion-specific evaluation of PCAT could predict ischemic coronary stenosis [36, 38] and provided incremental prognostic value for MACE assessment [39]. We found lesion-specific Rad-score was an independent predictor for MACE and showed incremental values over traditional risk factors in model comparison, which was consistent with the research [11] indicating CCTA-based fat radiomic profile improved risk prediction for adverse clinical events by radiomics analysis of RCA and left coronary artery. High-risk patients according to the optimum cutoff of Rad-score should proactively monitor the plaque progression and treat CAD in a timely manner, such as optimal medical treatment, even intensive drug therapy and revascularization therapy.

Radiomics features are generally recognized as quantitative biomarkers of heterogeneity as they are strongly related with the PCAT pathophysiological changes. Of the selected six features, three were three first-order features, one shape feature and 2 texture features. Among the significant radiomics features, texture homogeneity, intensity distribution and histogram features within PCAT were particularly crucial in predicting the MACE risk. An important reason is that an inflamed artery inhibits differentiation and lipid accumulation in PVAT pre-adipocytes, and induces fibrosis and microvascular remodeling followed by PCAT composition changes. Sphericity, a measure of the roundness of the shape of the PCAT region relative to a sphere, was also emphasized in the study, which reflected the regularity of the PCAT structure. The PCAT composition remodeling in inflamed artery, such as fibrous, vascularity, lipid-poor and more aqueous phase, leads to subtle structural irregularities that are indiscernible visually. Therefore, intensity, texture and shape features could be indications of these processes, which hint the development of coronary atherosclerosis and potential MACE risk.

In our study, CT-FFR was associated with MACE in the validation cohort, however CT-FFR was not independent predictor of MACE in the training cohort unexpectedly, which would affect the models’ construction and might be related with two reasons. First, lesion-specific Rad-score was determined by CT-FFR, which had effect on the predictive performance of CT-FFR in multivariate Cox regression. Second, more than a quarter of patients accepted planned revascularization therapy, whose risk might have been modulated by revascularization therapy. Models’ construction had great influence on model prediction performance, especially for the combined model, which might be the reason why the combined model had marginally improvement compared with the clinical radiomic model. Through comparisons among clinical model, clinical radiomics model and combined model, both lesion-specific Rad-score and CT-FFR had incremental value in prognostic performance. Combined model achieved the highest C-index and AUC, which demonstrated the advancement of combined model.

This present study has developed a comprehensive model that incorporates multiple CT imaging parameters and clinical risk factors to predict MACE risk in CAD patients. The combined model captures the probability of MACE risk in CAD patients and allows for a noninvasive method to recognize the high-risk patients proactively. The model can help clinicians to implement aggressive treatment to reduce cardiovascular burden and risk of adverse event due to CAD progression. The measurements of the CT imaging parameters in our study were fully automated or semi-automated, which greatly improved the repeatability of the study. Though our patients were included retrospectively, we collected comprehensive clinical information, measured a wide range of CT imaging parameters, and combined these data with radiomics features to acquire an efficient model.

The present study was object to several limitations. First, the study was a single-center study and there had no external validation, which could affect the robustness and generalization of the proposed model. Second, image acquisition was acquired from the same CT manufacturer in order to ensure the image uniformity, which needed to verify the generalization of the present findings in other manufacturers further. Meanwhile, different tube voltage because of adaptive scanning mode would have effect on the first-order features. Third, lesion-specific analysis of PCAT may not be applicable to patients without definite lesions in CCTA. Fourth, Target plaque in patients with multivessel disease was determined based on the lowest CT-FFR value, which might be not the plaque with the most inflammation. Last but not least, there might exist measuring error when different phases in CCTA images were selected.

In conclusion, lesion-specific Rad-score shows potential for MACE risk prediction. A comprehensive predictive model combining traditional clinical risk, Rad-score and CT-FFR has superior efficacy in predicting MACE in CAD patients. By detecting hemodynamically significant stenosis and inflammation, our study would be helpful for identifying high-risk patients to optimize risk management.

### Electronic supplementary material

Below is the link to the electronic supplementary material.

Supplementary Material 1

## Data Availability

The datasets during this study are not publicly available, but are available from the corresponding author on reasonable request.

## Abbreviations

CAD: Coronary artery disease
ACS: Acute coronary syndrome
CCTA: Coronary computed tomography angiography
PCATa: Pericoronary adipose tissue CT attenuation
MACE: Major adverse cardiovascular event
RCA: Right coronary artery
LAD: Left anterior descending artery
LCX: Left circumflex artery
CT-FFR: CT-derived fractional flow reserve
CACS: Coronary artery calcium score; HRP: High-risk plaque
SIS: Segment involvement score
LM: Left main coronary artery
