# Supplementary material for: Radiomics analysis of lesion-specific pericoronary adipose tissue to predict major adverse cardiovascular events in coronary artery disease
Source: BMC Med Imaging. 2024 Jun 17;24:150. doi: 10.1186/s12880-024-01325-1 (PMC11184685; doi:10.1186/s12880-024-01325-1)
Supplement: Supplementary file 1 — Supplementary Material 1 [file 12880_2024_1325_MOESM1_ESM.docx]

Supplementary material

**Radiomics analysis of lesion-specific pericoronary adipose tissue to predict major adverse cardiovascular events in coronary artery disease**

Supplementary Figures: 1

Supplementary Tables: 2

1. **Study population**

**1.1 Exclusion criteria**

The exclusion criteria were as follows: (1) patients without definite coronary plaque or stenosis; (2) poor image quality with 2 score or below on a 4-point Likert scale [1]. (3) patients with history of cardiac surgery, percutaneous coronary intervention (PCI) or coronary artery bypass grafting (CABG); (4) anomalous origin of coronary artery; (5) acute or chronic myocardial infarction (MI); (6) patients with serious life-threatening disease. A total of 608 consecutive coronary artery disease (CAD) patients were included (Fig S1).

**1.2 Training and validation cohorts**

Patients with major adverse cardiovascular events (MACE) were divided at a ratio of 7:3 with a random number table and patients without MACE were divided with the same way so as to balance the percentages of patients with MACE between the two cohorts. 70% of patients with and without MACE were assigned to the training cohort (n = 425, 102 MACE+, 323 MACE-) and the rest of 30% were included in the validation cohort (n = 183, 44 MACE+, 139 MACE-).


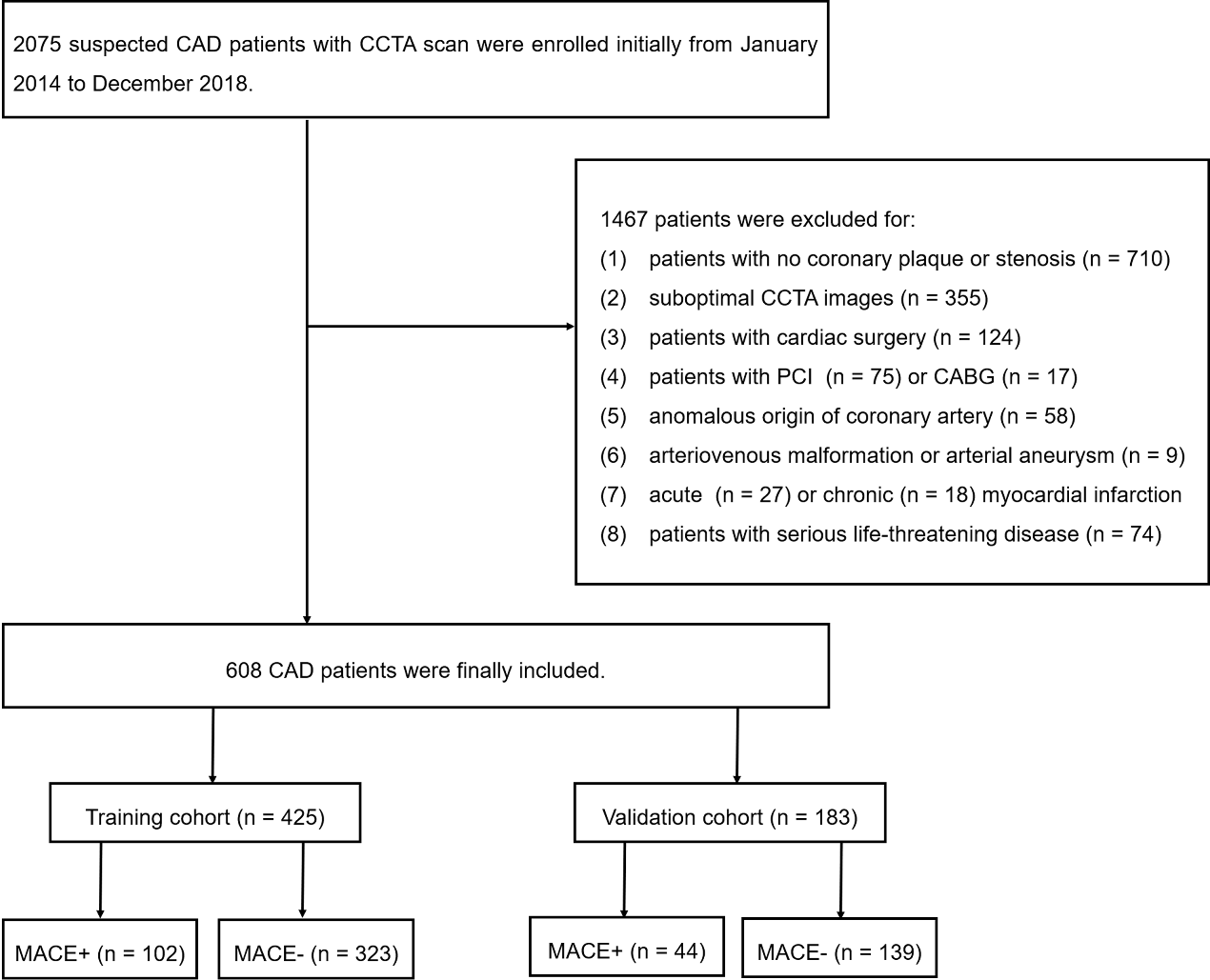


Figure S1 Flow diagram.

1. **Corornary CT angiography (CCTA) protocols**

All image acquisitions were performed with second-generation dual-source CT system (SOMATOM Definition Flash, Siemens Healthineers, Germany). Patients with heart rate ≧ 70 beats/min acquired oral beta-blockers. Every patient took sublingual nitroglycerin before CCTA scan. All patients underwent nonenhanced calcium score scan and CCTA scan (Table S1**)**. Adaptive auto electrocardiograph gating and smart arrhythmia management were used in CCTA scan. A bolus-tracking technology with a threshold of 220 Hounsfield units (HU) and a region of interest in the ascending aorta was used with 60 mL of iodinated contrast medium [Iodixanol (Nycomed, Norway) or Ultravist (Bayer, Germany)] administered at a flow rate of 5 ml/s.

Table S1 Scan parameters

| Scan parameters | | Training cohort (n=425) | Validation cohort  (n=183) | *p value* |
| --- | --- | --- | --- | --- |
| CCTA scan | |  |  |  |
|  | Tube voltage (KV) |  |  | 0.924 |
|  | 80 | 93 (21.9) | 42 (23.0) |  |
|  | 100 | 311 (73.2) | 133 (72.7) |  |
|  | 120 | 21 (4.9) | 8 (4.4) |  |
|  | Tube current (mAs) | 215 (187, 253) | 220 (195, 254) | 0.220 |
|  | Slice thickness (mm) | 0.6 | 0.6 | NA |
|  | Slice gap (mm) | 0.6 | 0.6 | NA |
|  | FOV (cm) | 21.2 | 21.2 | NA |
|  | Retrospective gating | Yes | Yes | NA |
|  | Scan trigger mode | Bolus tracking | Bolus tracking | NA |
|  | Contrast material | Iodixanol or Ultravist | Iodixanol or Ultravist | NA |
|  | Volume (ml) | 60 | 60 | NA |
|  | Iodine flux (g/s) | 1.6-1.85 | 1.6-1.85 | NA |
|  | CT value of aortic root (HU) | 569.02 (502.83, 678.28) | 586.27 (500.16, 693.03) | 0.795 |
|  | Noise | 25.59 (22.58, 30.84) | 26.05 (22.67, 31.51) | 0.429 |
|  | Estimated effective dose (ED) | 5.94 (4.61, 7.51) | 6.32 (4.78, 7.64) | 0.276 |
| Calcium score scan | |  |  |  |
|  | Tube voltage (KV) | 120 | | NA |
|  | Tube current (mAs) | 35 (23, 46) | 36 (22, 50) | 0.399 |
|  | Estimated effective dose (ED) | 1.80 (1.29, 2.39) | 1.83 (1.32, 2.55) | 0.430 |

CCTA, coronary computed tomographic angiography; FOV, field of view.

Iterative reconstruction was used to reconstruct CCTA images among the diastolic and systolic phases. CCTA analysis adopted the phase with optimal image quality and the diastolic phase was preferred when both were satisfied.

1. **Definition of MACE**

MACE included cardiovascular death, nonfatal MI, unplanned revascularization and hospitalization for unstable angina. Death cases consist of cardiovascular death, non-cardiovascular death, and undetermined cause of death. Cardiovascular death refers to death attributable to sudden cardiac death, acute MI, heart failure, stroke, cardiovascular procedure, cardiovascular hemorrhage, and other cardiovascular causes, such as pulmonary embolism or peripheral arterial disease [2]. Non-fatal MI is defined as acute myocardial injury with clinical evidence of acute MI and with detection of a rise and/or fall of cardiac troponin (cTn) values with at least 1 value above the 99th percentile upper reference limit (URL) and at least 1 of the following: (1) symptoms of MI; (2) new ischemic electrocardiogram (ECG) changes; (3) development of pathological Q waves; (4) imaging evidence of new loss of viable myocardium or new regional wall motion abnormality in a pattern consistent with an ischemic etiology; (5) identification of a coronary thrombus by angiography [3]. Unplanned revascularization is defined as revascularization at least 60 days after the coronary computed tomography angiography (CCTA) scan [4]. Unstable angina is defined as having newly developed or accelerating chest symptoms on exertion or rest angina, and the final diagnosis is myocardial ischemia with objective evidence but without elevation of cardiac biomarkers [5]. For patients who were lost during follow-up, the survival time was the interval between CCTA time and the last follow-up.

1. **Intra-observer and inter-observer reliability**

Table 2 Intra-observer and inter-observer reliability

| Variables | Intra-observer ICC  (n = 50) | *p* |  | Inter-observer ICC  (n = 50) | *p* |
| --- | --- | --- | --- | --- | --- |
| CACS | 0.926 (0.873-0.957) | ＜0.001 |  | 0.970 (0.947-0.983) | ＜0.001 |
| SIS | 0.979 (0.964-0.988) | ＜0.001 |  | 0.975 (0.957-0.986) | ＜0.001 |
| Plaque volume | 0.965 (0.938-0.980) | ＜0.001 |  | 0.970 (0.947-0.983) | ＜0.001 |
| CT-FFR | 0.930 (0.863-0.962) | ＜0.001 |  | 0.918 (0.844-0.955) | ＜0.001 |
|  | Intra-observer Kappa  (n = 50) | *p* |  | Inter-observer Kappa  (n = 50) | *p* |
| Diameter stenosis | 0.823 | ＜0.001 |  | 0.820 | ＜0.001 |
| High-risk plaque | 0.878 | ＜0.001 |  | 0.840 | ＜0.001 |
| Low attenuation | 0.874 | ＜0.001 |  | 0.831 | ＜0.001 |
| Spotty calcification | 0.959 | ＜0.001 |  | 0.880 | ＜0.001 |
| Napkin ring sign | 0.715 | ＜0.001 |  | 0.648 | ＜0.002 |
| Positive remodeling | 0.802 | ＜0.001 |  | 0.758 | ＜0.001 |

ICC, intraclass correlation coefficient; CACS, coronary artery calcium score, SIS, segment involvement score; CT-FFR, computed tomography derived fractional flow reserve.

Supplemental references

1. Dai X, Yu M, Pan J, Lu Z, Shen C, Wang Y, et al. Image quality and diagnostic accuracy of coronary CT angiography derived from low-dose dynamic CT myocardial perfusion: a feasibility study with comparison to invasive coronary angiography. Eur Radiol. 2019;29:4349-4356.

2. Hicks KA, Tcheng JE, Bozkurt B, Chaitman BR, Cutlip DE, Farb A, et al. 2014 ACC/AHA Key Data Elements and Definitions for Cardiovascular Endpoint Events in Clinical Trials: A Report of the American College of Cardiology/American Heart Association Task Force on Clinical Data Standards (Writing Committee to Develop Cardiovascular Endpoints Data Standards). J Am Coll Cardiol. 2015;66:403-469.

3. Thygesen K, Alpert JS, Jaffe AS, Chaitman BR, Bax JJ, Morrow DA, et al. Fourth Universal Definition of Myocardial Infarction (2018). Circulation. 2018;138:e618-e651.

4. Sen S, Ahmad Y, Dehbi HM, Howard JP, Iglesias JF, Al-Lamee R, et al. Clinical Events After Deferral of LAD Revascularization Following Physiological Coronary Assessment. J Am Coll Cardiol. 2019;73:444-453.

5. Zhou F, Chen Q, Luo X, Cao W, Li Z, Zhang B, et al. Prognostic Value of Coronary CT Angiography-Derived Fractional Flow Reserve in Non-obstructive Coronary Artery Disease: A Prospective Multicenter Observational Study. Frontiers in Cardiovascular Medicine. 2022;8.
